# Differences in the prefrontal cortex responses of healthy young men performing either water-based or land-based exercise at light to moderate intensity

**DOI:** 10.1007/s00221-023-06583-z

**Published:** 2023-03-21

**Authors:** Tatsuya Hashitomi, Daisuke Hoshi, Marina Fukuie, Takashi Tarumi, Jun Sugawara, Koichi Watanabe

**Affiliations:** 1 Doctoral Program in Sports Medicine, Graduate School of Comprehensive Human Sciences, University of Tsukuba, Tsukuba, Ibaraki Japan; 2 Human Informatics and Interaction Research Institute, National Institute of Advanced Industrial Science and Technology, Tsukuba, Ibaraki Japan; 3 Faculty of Health and Sports Sciences, University of Tsukuba, 1-1-1, Tennodai, Tsukuba, Ibaraki 305-8577 Japan

**Keywords:** Water cycling, Water immersion, Functional near-infrared spectroscopy, Prefrontal cortex, Oxy-Hb

## Abstract

Cerebral blood flow increases more during water-based exercise than land-based exercise owing to the effects of end-tidal CO_2_ (PETCO_2_) and mean arterial pressure (MAP) changes due to water immersion. However, it is unclear whether oxygenated hemoglobin (oxy-Hb) concentrations in the prefrontal cortex (PFC) are increased more by water-based or land-based exercise. We hypothesized that oxy-Hb concentrations in the PFC are higher during water-based exercise than land-based exercise when the exercise intensity is matched. To test this hypothesis, 10 healthy participants (age: 24.2 ± 1.7 years; height: 1.75 ± 0.04 m; weight: 69.5 ± 5.2 kg) performed light- to moderate-intensity cycling exercise in water (water-based cycling (WC); chest-high water at 30 °C) and on land (LC). Stroke volume, cardio output, heart rate, MAP, respiratory rate, PETCO_2_, and oxy-Hb in the PFC were assessed during 15 min of exercise, with exercise intensity increased every 5 min. Both WC and LC significantly increased oxy-Hb concentrations in the PFC as exercise intensity was increased (intensity effect: *p* < 0.001). There was no significant difference in oxy-Hb concentrations during WC and LC in most prefrontal areas, although significant differences were found in areas corresponding to the left dorsolateral PFC (exercise effect: *p* < 0.001). Thus, WC and LC increase oxy-Hb concentrations in the PFC in a similar manner with increasing exercise intensity, but part of the PFC exhibits enhanced oxy-Hb levels during WC. The neural response of the PFC may differ during water-based and land-based exercise owing to differences in external information associated with water immersion.

## Introduction

Exercise has beneficial effects on cerebral function (Hillman et al. 2008; Guadagni et al. 2020; Voss et al. 2013). Previous studies involving transcranial Doppler examinations and oxygenated hemoglobin (oxy-Hb) concentration measurements in the prefrontal cortex (PFC) revealed that cerebral blood flow (CBF) velocity and increases under light- and moderate-intensity aerobic exercise (Rooks et al. 2010; Smith and Ainslie 2017). However, it is difficult for elderly individuals with declining physical function and/or joint disorders to perform higher intensity exercise, and high exercise intensity can inhibit exercise continuity and motivation (Ekkekakis et al. 2011; Zenko et al. 2016). Thus, middle- and old-aged individuals often prefer water-based exercise because water resistance lowers the risk of falling during exercise and water buoyancy reduces the gravity-induced strain on joints.

CBF is regulated by a coordinated combination of many different systems (Girouard et al. 2006; Paulson et al. 1990). Of these systems, the chemical, cardiovascular, metabolic, and neural systems are well documented, with the main factors affecting CBF regulation cited as cardio output (CO), arterial blood pressure, and carbon dioxide (Querido and Sheel 2007). Immersion in water and water-based exercise induce different physiological responses than being on land and land-based exercise, and the variables typically involved in CBF regulation have been reported to differ during water immersion and water-based exercise. For example, water immersion increases hydrostatic pressure, stroke volume (SV), and CO but decreases heart rate (HR) (Christie et al. 1990; Garzon et al. 2015; Park et al. 1999). End-tidal carbon dioxide (PETCO_2_) at rest has also been shown to increase during water immersion (Sackett et al. 2017).

Several studies have reported CBF responses during water immersion and water exercise. For instance, water immersion at the femur level increases middle and posterior cerebral artery velocities (Carter et al. 2014). In addition, during water immersion at the right atrium level, light-intensity water-based stepping exercise increases middle and posterior cerebral artery velocities relative to the respective velocities achieved during land-based stepping exercise. The increases in CBF during water immersion at rest and exercise are related to increases in mean arterial pressure (MAP) and PETCO_2_, suggesting that these two variables contribute to the augmentation of CBF during water-based exercise (Pugh et al. 2015).

Although studies have shown that water-based exercise increases CBF more than land-based exercise, it is not clear how oxy-Hb levels, which reflect neural activity, change in the PFC during water-based exercise. Previous studies have reported that changes in oxy-Hb are related to changes in PETCO_2_ and hemodynamics (Rooks et al. 2010). Therefore, as a first step to accumulating basic data on the effects of water-based exercise on brain function, we focused on young people. The purpose of the present study was to determine whether oxy-Hb concentrations of the PFC in young adults at light- to moderate-intensity exercise are increased more by water-based exercise or land-based exercise when exercise intensity is matched. We hypothesized that the level of prefrontal oxy-Hb is higher during water-based exercise than land-based exercise owing to the hemodynamic and PETCO_2_ changes associated with water immersion.

## Methods

### Participants

Ten healthy participants were enrolled in this study (age: 24.2 ± 1.7 years; height: 1.75 ± 0.04 m; weight: 69.5 ± 5.2 kg; body mass index: 22.7 ± 1.1 kg/m^2^). All participants were nonsmokers with no history of cardiovascular, musculoskeletal, or metabolic disease or any contraindications to exercise. None of the participants reported taking any medication. This study was approved by the Institutional Ethics committee of Tsukuba University and was performed in accordance with the latest version of the Declaration of Helsinki. Written informed consent was obtained from all participants after they were provided with a complete description of the study.

### Experimental protocol

Each participant visited the laboratory three times and one set of measurements were taken per visit in the following order: peak oxygen consumption (VO_2_peak) measurement, water-based cycling (WC) test, and land-based cycling (LC) test. The participants were instructed to fast for a minimum of 3 h and refrain from vigorous exercise, alcohol consumption, and caffeine intake at least 24 h before each visit. All visits were separated by at least 72 h. We needed to match exercise intensity between WC and LC, but when WC and LC were performed in a random order, it was difficult to match oxygen uptake (VO_2_) under both conditions. Therefore, based on the VO_2_ at each stage of WC, the VO_2_ and ergometer workload (W) values measured in the first VO_2_peak measurement were applied to the workload at each stage of LC.

### VO_2_peak measurement

We defined the average value for 30 s just before the end of the incremental cycling exercise as the VO_2_peak. In the test, a recumbent bicycle (Corival Recumbent, Lobe, BV, Netherlands) and expiratory gas analyzer (Aero Monitor, MINATO, Osaka, Japan) were used to assess oxygen uptake during exercise. The ergometric workload was started at 60 W and increased by 20 W every 2 min. At the point where the phase of cycling at 160 W for 2 min was completed, the workload was increased by 10 W until two of the following criteria were met: (1) the participant’s heart rate reached the 95% heart rate reserve, (2) the participant’s respiratory quotient was > 1.2, (3) the participant was unable to maintain cycling at 60 ± 5 rpm, and/or (4) the rate of perceived exercise intensity was > 18.

### Cycling procedure

**Fig. 1:**
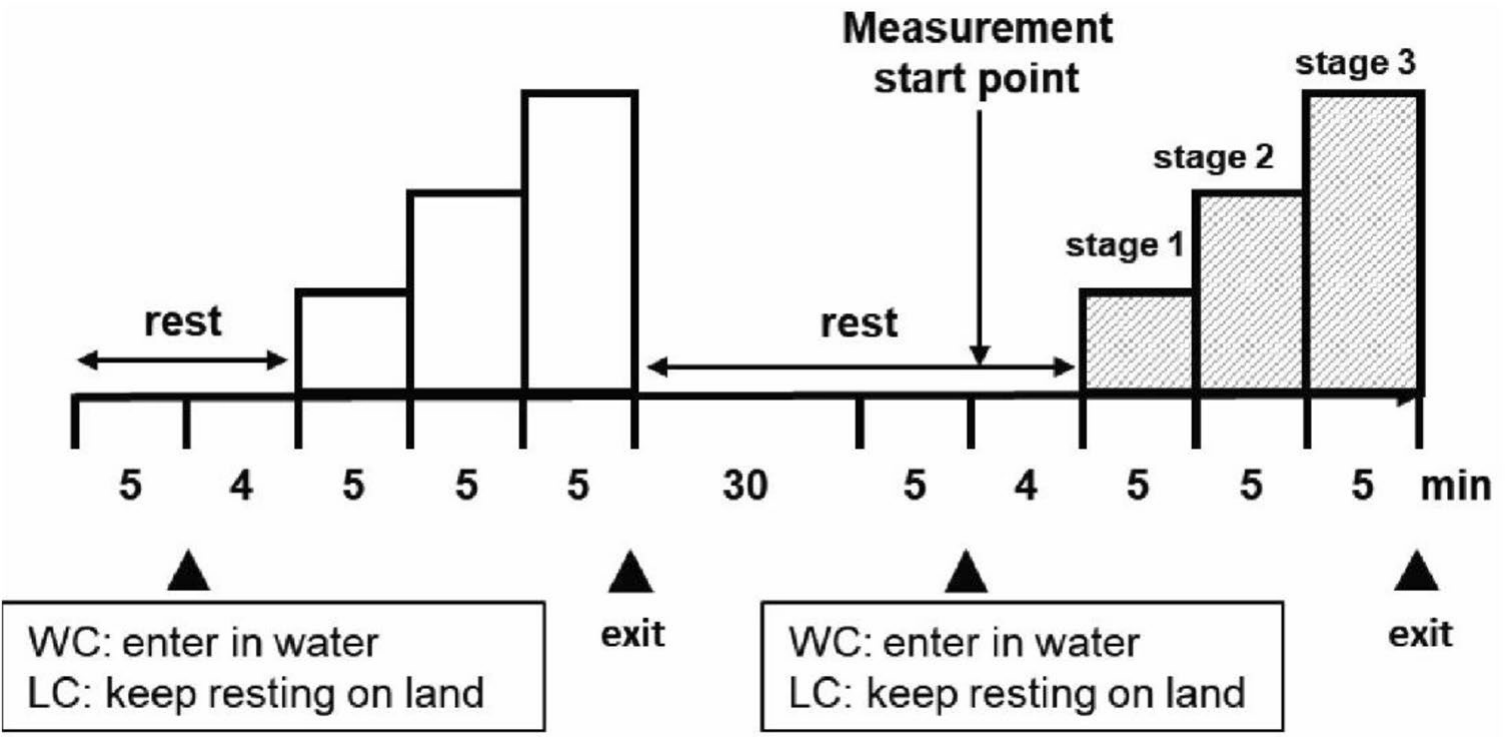
Cycling procedure. *WC* cycling in water, *LC* cycling on land

First, the participants practiced the cycling exercise for 15 min with no load and an increase in cadence by 15 rpm every 5 min in each test environment to acclimatize to the cycling exercise under both conditions. Under the WC condition, the participants wore swimwear and rested in a seated position for 5 min out of the water, after which they were submersed in water at 31–32 ℃ to the xiphoid appendix level and sat for 4 min. Since the temperature normally used in ordinary heated swimming pools is set slightly lower than the neutral water temperature of 34 °C, this temperature range was also set for this experiment. Subsequently, the participants conducted 15 min of cycling exercise using a water-based stationary semi-recumbent bicycle (Hydrorecline, H3Oz Company, Italy). The cycling exercise began at 30 rpm, and the cadence was increased by 15 rpm every 5 min (stages 1–3, respectively). Participants maintained the cadence according to the sound of a metronome. To increase pedaling resistance, three plastic paddles (7.0 × 22.0 × 0.5 cm) were placed on the rear wheel. During the exercise test, the participants’ cardiorespiratory and hemodynamic variables were monitored. Under the LC condition, the participants rested in a seated position on the land at room temperature (25–26 ℃) for 5 min, after which they rested for another 4 min to match the WC procedure. After resting, the participants conducted 15 min of cycling exercise. Using a recumbent bicycle (Corival Recumbent), the participants underwent VO_2_-matched cycling exercise (with the wheel-load adjusted electrically every 5 min), and their cardiorespiratory and hemodynamic variables were measured. During the exercise, we instructed the participants to maintain cadence at 60 rpm (Fig. 1).

### Functional near-infrared spectroscopy measurement

We used an 8-channel functional near-infrared spectroscopy (fNIRS) system (OctaMon, Artinis Medical Systems, Netherlands) to measure changes in oxy-Hb levels in the PFC. For fNIRS probe placement, the international 10–20 system was used to ensure consistency. First, we identified the Cz position from the middle point of the nasion and inion and the middle point of the left and right preauricular point. Second, the areas 20% left and right toward the left and right preauricular point from the Cz were set as C3 and C4, respectively. Third, the area 5 cm sagittally from C3 and C4 was covered by CH1 and CH7 of fNIRS, respectively; hence, the left and right dorsolateral PFC (DLPFC) areas were covered with CH1 and CH7, respectively. All parameters were measured using 765 and 855 nm penetrating wavelengths with an emitter to optode distance of 3.5 cm, resulting in a penetrated tissue depth of approximately 1.50–1.75 cm. The 8-channel system recorded the hemoglobin signals at 10 Hz. MATLAB was used to analyze the fNIRS data. First, noise (from head movement) was automatically detected and excluded from the acquired data. Second, timeline data were preprocessed with a bandpass filter using cut-off frequencies of 0.04 Hz to remove baseline drift and 0.7 Hz to filter out heartbeat pulsations. Third, 20 s just before the end of rest before exercise was set as the baseline, and the baseline was subtracted from the oxy-Hb level measured during cycling for 15 min. The oxy-Hb volume in the last 20 s of each 5-min exercise stage was considered the oxy-Hb volume in each stage. Each channel (CH1–8) and all channel averages (All-CH) were analyzed.

### Systemic hemodynamics variables

Throughout the experiment, the digital arterial pressure waveform was measured from the participant’s middle finger using a noninvasive blood pressure recording device (INL382, AD Instrument, Colorado Springs, CO, USA), and the data were stored on a computer at 1 kHz of the sampling rate using a data acquisition system (PowerLab, AD Instrument, Colorado Springs, CO, USA) interfaced with a computer running dedicated acquisition and analysis software (LabChart ver.8, AD Instrument). We asked participants to keep their right hand at heart level throughout the rest and exercise periods (placed on a side table outside the bathtub under the WC condition). HR and blood pressure were calculated using the LabChart analysis software, and SV and CO were estimated using the Modelflow-based add-on program for this software.

### Breathing pattern

Respiratory rate (RR) and PETCO_2_ were measured or calculated using the expiratory gas analyzer system (Aero monitor, MINATO, Osaka, Japan) during 4-min rest under each resting condition and 15 min of cycling exercise under each exercise condition. These variables were measured using the breath-by-breath method and averaged over the last 20 s of 4-min rest and each 5-min stage of exercise. Data collection was standardized. The expiratory gas analyzer was calibrated before each experiment according to the manufacturer’s instructions.

### Eardrum temperature

The body temperature of each participant was measured according to their right eardrum temperature at rest and at 4 min of each exercise stage.

### Estimation of sample size

Sample size and statical power were calculated using the G*Power software. The following parameters were considered according to previous research (Cohen 1992): F test (analysis of variance [ANOVA]); effect size: 0.8; alpha level: 0.05; statistical power: 0.8; number of groups: 2 (WC and LC); number of measurement periods: 3 (stage1, stage2, stage3); and number of participants who refused to participants: 2. As a result, the size of the total sample was 12.

### Statistical analysis

Statistical analyses were performed using the Statistical Packages for Social Sciences for Windows version 28 (IBM Inc). Statistical significance was set at *p* < 0.05. Two-factor repeated-measures ANOVA was used to analyze the exercise factor (WC and LC) and stage factor. Stage factors were classified as follows for each variable: oxy-Hb level: stage 1, stage 2, and stage 3; other parameters: rest, stage 1, stage 2, and stage 3. The change per stage was calculated, and correlation coefficients were used to determine the strength of relationships between the change in oxy-Hb levels (Δoxy-Hb) and the change in other variables (i.e., ΔMAP, ΔRR, ΔPETCO_2_, and ΔCO) at each stage (i.e., change from rest to stage 1: 1st stage; change from stage 1 to stage 2: 2nd stage; change from stage 2 to stage 3: 3rd stage).

## Results

The WC and LC exercise bouts were successfully matched for VO_2_ (exercise, *p* < 0.001; stage and exercise–stage interaction, *p* > 0.05, respectively). %VO_2_peak changed significantly according to stage (*p* < 0.001) but not exercise (*p* > 0.05), nor was their interaction effect significant (*p* > 0.05). Exercise intensity was about 8% VO_2_peak at stage 1, 17% VO_2_peak at stage 2, and 47% VO_2_peak at stage 3 under both conditions, in which stages 1 and 2 involved light-intensity exercise and stage 3 involved moderate-intensity exercise. The rate of perceived exertion (RPE) changed significantly according to stage (*p* < 0.001) but not exercise (*p* > 0.05), nor was their interaction effect significant (*p* > 0.05) (Table 1). HR increased significantly throughout the stages of both protocols (*p* < 0.001) but did not differ significantly according to exercise (*p* > 0.05), nor was the exercise–stage interaction effect significant (*p* > 0.05) (Table 2).

**Table 1:** Changes in VO_2_, body temperature, RPE according to exercise and intensity

	Rest	Stage 1	Stage 2	Stage 3	*P* value		
	Mean ± SD	Mean ± SD	Mean ± SD	Mean ± SD	Intensity	Environment	Interaction
Cadence, rpm							
WC	0	30	45	60			
LC	0	60	60	60			
Workloads, watt							
WC	–	–	–	–			
LC	0 ± 0	16.5 ± 4	47.2 ± 5.4	104.4 ± 14.5			
RPE							
WC	6.1 ± 0.6	7.3 ± 1.2	9.0 ± 1.2	12.3 ± 1.2	< 0.001	0.522	0.351
LC	6.1 ± 1.0	8.0 ± 1.0	10.0 ± 1.2	121.1 ± 1.7			
VO_2_, mL/kg/min							
WC	3.3 ± 0.5	5.8 ± 0.5	9.0 ± 0.8	16.6 ± 1.9	< 0.001	0.621	0.662
LC	3.2 ± 0.4	6.1 ± 0.7	9.4 ± 0.8	16.9 ± 9.4			
%VO_2peak_, %							
WC	9.3 ± 1.2	16.4 ± 2.6	25.5 ± 3.9	46.9 ± 7.9	< 0.001	0.779	0.734
LC	8.9 ± 1.2	17.2 ± 3.0	26.0 ± 3.0	47.1 ± 8.1			
Body temperature, ℃							
WC	35.3 ± 0.6	35.4 ± 0.6	35.4 ± 0.5	35.5 ± 0.5	0.037	0.695	0.405
LC	35.2 ± 0.4	35.3 ± 0.4	35.4 ± 0.3	35.5 ± 0.4			

*SD* standard deviation, *WC* cycling in water, *LC* cycling on land, *RPE* rate of perceived exertion, *VO*_*2*_ oxygen uptake, *%VO*_*2*_*peak* peak oxygen consumption

**Table 2:** Systemic hemodynamics at rest and during exercise

	Rest	Stage 1	Stage 2	Stage 3	*P* value		
	Mean ± SD	Mean ± SD	Mean ± SD	Mean ± SD	Intensity	Environment	Interaction
HR, bpm							
WC	58.2 ± 7.8	71.3 ± 6.7	81.3 ± 11.5	107.2 ± 19.6	< 0.001	0.246	0.499
LC	67.5 ± 11.1	73.4 ± 11.8	87.9 ± 11.1	114.4 ± 17.7			
SBP, mmHg							
WC	110.5 ± 7.2	113.4 ± 6.7	118.7 ± 6.6	136.9 ± 6.4	< 0.001	< 0.001	0.747
LC	118.7 ± 7.8	78 ± 8	76 ± 9	147.1 ± 8.1			
DBP, mmHg							
WC	71.5 ± 5.3	70.7 ± 5.2	70.6 ± 4.2	70.9 ± 5.3	0.707	0.006	0.889
LC	78.4 ± 6.1	78.3 ± 3.5	76.8 ± 6.0	77.6 ± 3.5			
MAP, mmHg							
WC	84.4 ± 4.9	85.0 ± 4.8	86.6 ± 2.8	92.9 ± 4.8	0.006	0.707	0.889
LC	91.8 ± 5.6	94.1 ± 4.0	94.3 ± 5.6	101.2 ± 6.7			

*SD* standard deviation, *WC* cycling in water, *LC* cycling on land, *HR* heart rate, *SBP* systolic blood pressure, *DBP* diastolic blood pressure, *MAP* mean arterial pressure

### Oxy-Hb levels

Two-way repeated-measures ANOVA revealed a significant main effect of stage in each CH and All-CH (*p* < 0.001, respectively). However, exercise was a significant factor for CH7 only (*p* = 0.039), and the exercise–stage interaction effect was not significant for each CH and All-CH (*p* > 0.05) (Table 3).

**Table 3:** Change in oxy-Hb concentrations in the prefrontal cortex in each channel at each stage of exercise

	Stage 1	Stage 2	Stage 3	*P* value		
	Mean ± SD	Mean ± SD	Mean ± SD	Intensity	Exercise	Interaction
CH1						
WC	0.23 ± 1.6	1.86 ± 2.0	5.01 ± 2.9	< 0.001	0.437	0.766
LC	− 0.80 ± 1.7	1.12 ± 1.5	4.51 ± 3.7			
CH2						
WC	0.42 ± 1.1	1.46 ± 1.6	4.78 ± 2.6	< 0.001	0.833	0.972
LC	0.40 ± 0.7	1.35 ± 1.8	4.62 ± 3.3			
CH3						
WC	0.20 ± 1.4	2.01 ± 1.6	5.62 ± 3.4	< 0.001	0.378	0.833
LC	− 0.25 ± 0.7	1.18 ± 1.9	4.66 ± 3.6			
CH4						
WC	0.59 ± 1.3	1.78 ± 1.7	4.63 ± 3.1	< 0.001	0.259	0.788
LC	− 0.26 ± 0.9	0.99 ± 2.0	3.38 ± 2.6			
CH5						
WC	− 0.08 ± 1.6	2.31 ± 1.9	5.50 ± 3.8	< 0.001	0.067	0.392
LC	− 0.37 ± 0.7	0.51 ± 1.4	3.78 ± 3.0			
CH6						
WC	− 0.44 ± 1.1	1.05 ± 1.5	3.92 ± 2.5	< 0.001	0.553	0.665
LC	− 0.45 ± 0.9	0.2 ± 1.4	3.74 ± 3.2			
CH7						
WC	− 0.46 ± 1.2	3.03 ± 1.9	7.17 ± 3.7	< 0.001	0.039	0.234
LC	− 0.71 ± 1.0	0.71 ± 0.8	4.81 ± 3.5			
CH8						
WC	− 0.37 ± 0.6	1.91 ± 0.8	5.54 ± 1.2	< 0.001	0.09	0.086
LC	− 0.23 ± 0.2	− 0.17 ± 0.4	2.91 ± 0.8			
ALL CH						
WC	0.01 ± 1.0	1.84 ± 1.4	5.32 ± 3.0	< 0.001	0.182	0.522
LC	0.34 ± 0.6	0.72 ± 1.2	4.0 ± 2.8			

*SD* standard deviation, *WC* cycling in water, *LC* cycling on land, *CH* each channel for fNIRS

### SV and CO

**Fig. 2:**
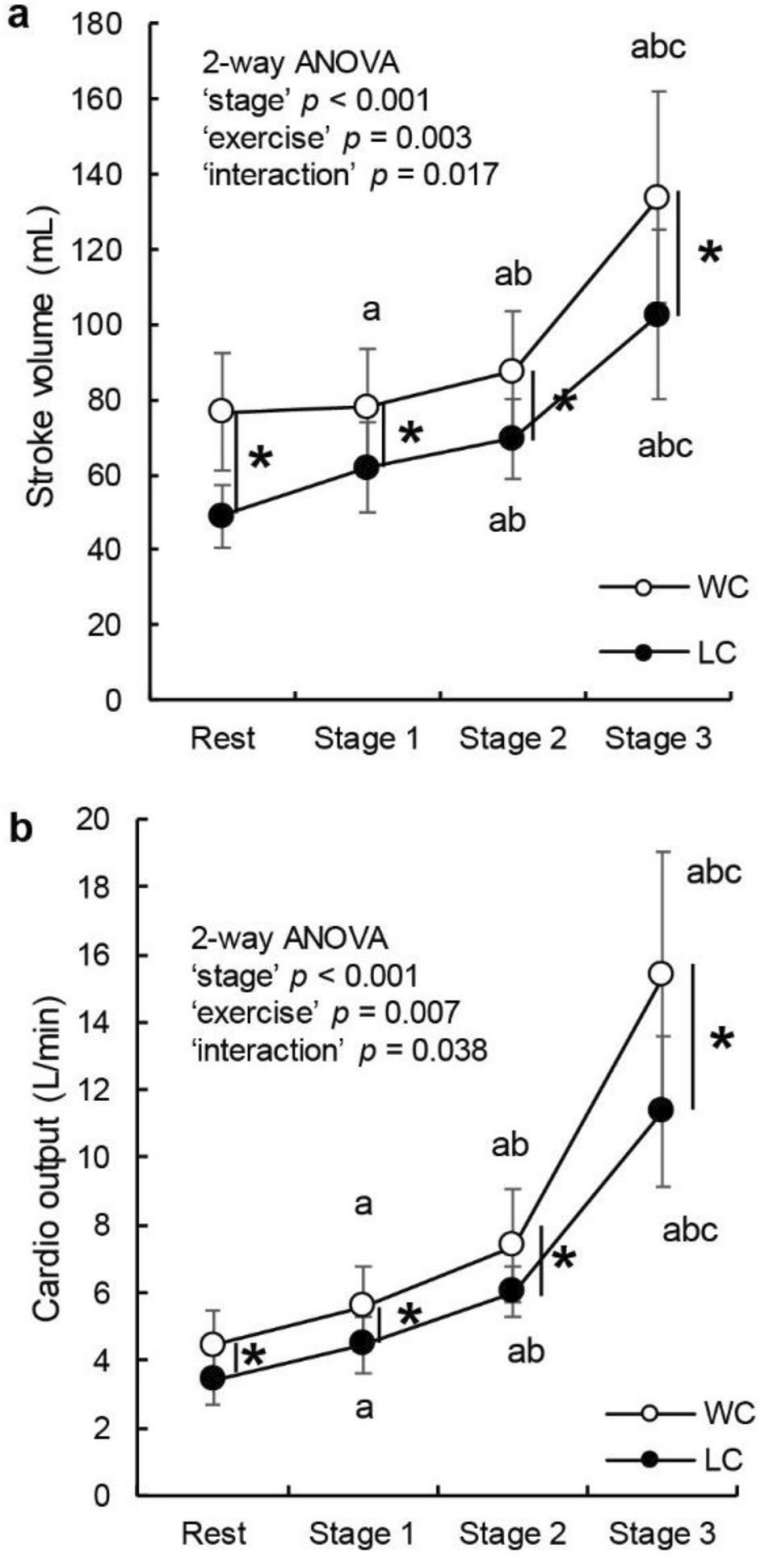
Change in stroke volume (mL) (**A**) and cardio output (L/min) (**B**) at rest and each exercise stage. White (○) and black (●) circles indicate WC and LC, respectively. Error bars represent SD. *P* values according to two-way ANOVA are presented (“stage”, “exercise”, and “interaction”). *P < 0.05, WC vs. LC. a, b, and c indicate differences from rest, stage 1, and stage 2, respectively

For SV and CO measurements, two-way repeated-measures ANOVA revealed a significant main effect of stage (*p* < 0.001, respectively), exercise (*p* = 0.003 and *p* = 0.007, respectively), and their interaction (*p* = 0.007 and *p* = 0.038, respectively). Post hoc tests revealed that SV and CO were significantly higher under WC than under LC at each stage (Fig. 2).

### PETCO_2_ and RR

**Fig. 3:**
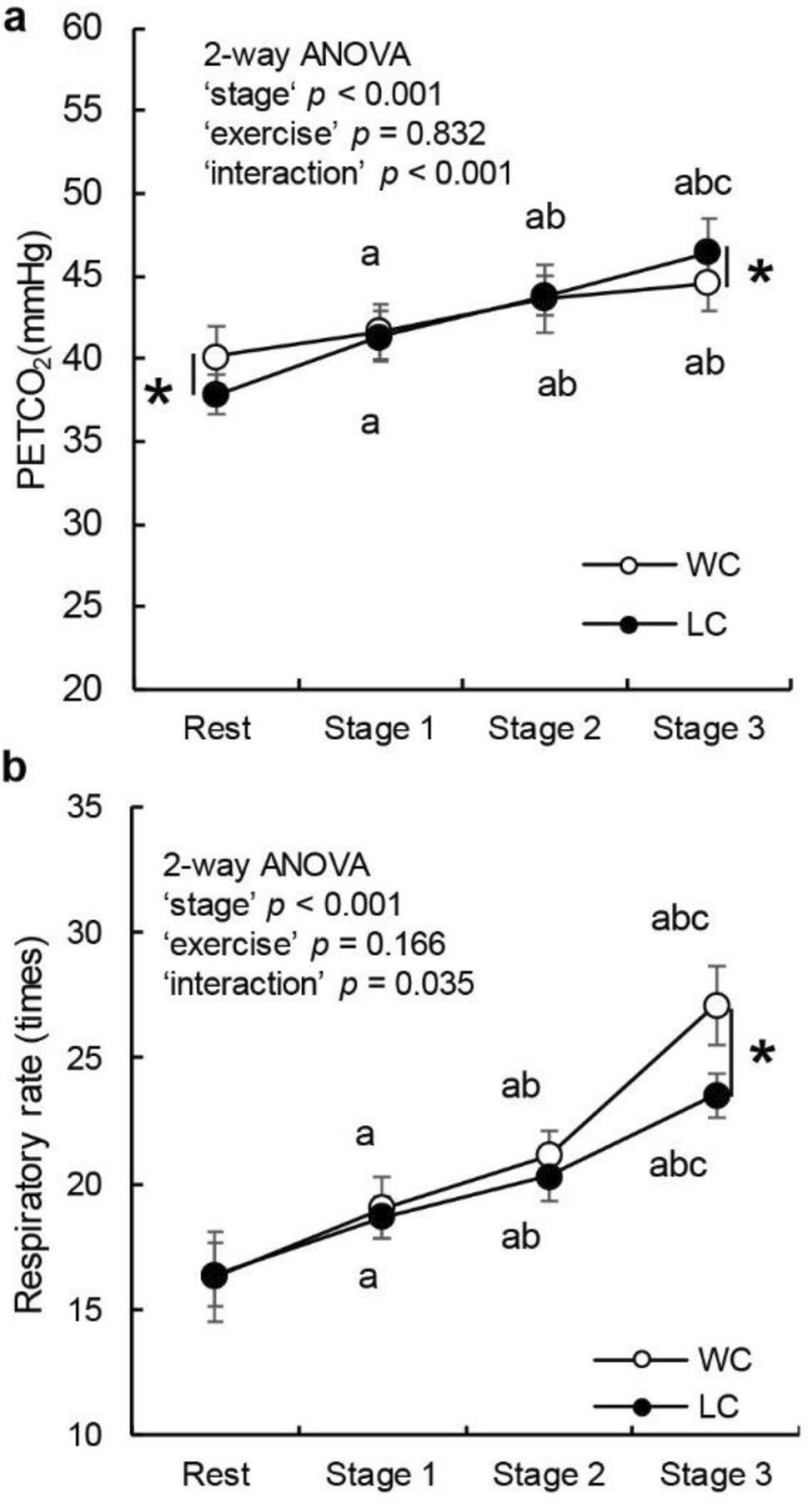
Change in end-tidal carbon dioxide (mmHg) (**A**) and respiratory rate (times) (**B**) at rest and each stage of exercise. White (○) and black (●) circles indicate WC and LC, respectively. Error bars represent SD. *P* values according to two-way ANOVA are presented (“stage”, “exercise”, and “interaction”). *P < 0.05, WC vs. LC. a, b, and c indicate differences from rest, stage 1, and stage 2, respectively

Two-way repeated-measures ANOVA revealed that PETCO_2_ changed significantly according to stage (*p* < 0.001) and exercise (*p* = 0.832), and their interaction effect was significant (*p* < 0.001). Post-hoc tests revealed that PETCO_2_ was higher at rest under WC than under LC but higher at stage 3 under LC than under WC. RR changed significantly according to stage (*p* < 0.001) and exercise (*p* = 0.166), and their interaction effect was significant (*p* = 0.035). Post-hoc tests revealed that RR was higher at stage 3 under WC than under LC (Fig. 3).

### Blood pressure

Two-way repeated-measures ANOVA revealed that systolic blood pressure changed significantly according to stage and exercise (*p* < 0.001, respectively), but their interaction effect was not significant (*p* = 0.747). Diastolic blood pressure did not change significantly with stage nor was the exercise–stage interaction effect significant (*p* > 0.05, respectively), although diastolic blood pressure changed significantly according to exercise (*p* = 0.006). MAP changed significantly according to stage (*p* = 0.006), but their exercise and interaction effect was not significant (*p* = 0.707, *p* = 0.889, respectively) (Table 2).

### Body temperature

Body temperature did not differ according to stage and exercise, and their interaction effect was not significant (*p* > 0.05, respectively).

### Correlation analysis for Δoxy-Hb

According to Pearson product-moment correlation coefficients, in the 1st stage, oxy-Hb under WC was correlated with PETCO_2_ (*r* = 0.51) and RR (*r* = 0.76), while oxy-Hb under LC was correlated with CO (*r* = − 0.80) and RR (*r* = 0.7). In the 2nd stage, oxy-Hb under WC was correlated with CO (− 0.44), PETCO_2_ (*r* = 0.34), and RR (*r* = − 0.66) while oxy-Hb under LC was correlated with CO (*r* = 0.40). In the 3rd stage, oxy-Hb under WC was correlated with RR (*r* = 0.5) while oxy-Hb under LC was correlated with CO (*r* = 0.50) and MAP (*r* = − 0.39).

## Discussion

In the present study, we found that oxy-Hb levels increased as the intensity of exercise was increased under both WC and LC conditions; however, oxy-Hb levels were higher under WC than LC in some regions of the PFC.

The WC and LC exercises at light to moderate intensity were successfully matched for VO_2_, indicating that the exercise protocols were similar in terms of relative intensity among participants. Regarding fNIRS data, we found no changes in tympanic membrane temperature during exercise and no difference in this temperature under WC and LC conditions, suggesting that the effect of oxy-hb on body temperature changes was minimal in both conditions. Previous studies on land-based exercise have found that incremental exercise increases the oxy-Hb concentration in the PFC at the light to high-intensity stage and decreases this concentration at the very high-intensity stage (Garzon et al. 2015). To the best of our knowledge, it was not known whether light- and moderate-intensity water-based exercise enhance the oxy-Hb concentration in the PFC. Thus, we show for the first time that oxy-Hb levels increase in the PFC under light to moderate exercise intensity in both WC and LC protocols; moreover, the oxy-Hb levels did not differ significantly between the WC and LC conditions in most CH at each stage. The mechanisms underlying the increase in oxy-Hb levels according to increasing exercise intensity likely differ between WC and LC. For example, LC showed correlations with CO in all stages, whereas WC showed correlations with RR and PETCO_2_ in each stage (Table 4).

**Table 4:** Correlations between Δoxy-Hb and other variables

	WC	LC
1st stage		
PETCO_2_	0.51	− 0.26
RR	0.76*	0.70*
CO	− 0.14	− 0.80**
MAP	0.27	0.28
2nd stage		
PETCO_2_	0.34	0.07
RR	− 0.66*	0.17
CO	− 0.44	0.40
MAP	0.07	− 0.15
3rd stage		
PETCO_2_	− 0.24	− 0.05
RR	0.51	− 0.02
CO	0.27	0.50
MAP	− 0.21	− 0.39

*WC* cycling in water, *LC* cycling on land, *PETCO*_*2*_ end-tidal carbon dioxide, *RR* respiratory rate, *CO* cardio output, *MAP* mean arterial pressure

*p < 0.05, **p < 0.01

The parameters related to an increase or decrease in CBF differed between the WC and LC conditions. First, CO was higher under WC than LC at each stage of exercise. Previous studies have reported a decrease in HR and increases in SV and CO due to water immersion (Christie et al. 1990; Garzon et al. 2015; Park et al. 1999); in the present study, both SV and CO were higher under WC than LC both at rest and during exercise. Additionally, HR tended to be lower under WC than LC, although not significantly lower. Thus, the participants exhibited physiological responses to water immersion in the present study. Nevertheless, the increase in CO during water immersion did not have a significant effect on the oxy-Hb concentration in the PFC. MAP tended to be higher in LC than in WC, but the difference was not significant. Also, ΔMAP and Δoxy-Hb showed no significant correlation. However, fluctuations in MAP during exercise have been reported to affect CBF (Smith and Ainslie 2017). Previous studies have also reported that light- and moderate-intensity exercise influenced a positive correlation between oxy-Hb and MAP (Tsubaki et al. 2016). Therefore, the changes in oxy-Hb of LC in this study may be more influenced by the MAP than the WC. At rest, PETCO_2_ increases during water immersion (Sackett et al. 2017). In the present study, RR did not differ between WC and LC, whereas PETCO_2_ was higher under WC than under LC at rest. The effect of water pressure likely caused spontaneous or voluntary hypoventilation, resulting in higher PETCO_2_. However, neither RR nor PETCO_2_ differed between the WC and LC conditions at the light-intensity stage of exercise; conversely, at the moderate-intensity stage of exercise, RR was higher under WC than LC, whereas PETCO_2_ was lower under WC than LC. Hyperventilation leads to lower PETCO_2_, and the increase in RR may have resulted in a plateau, if not a decrease, in PETCO_2_ that led to the observed reduction under WC (Pendergast et al. 2015). Ventilation has been suggested as a factor that affects CBF (Querido and Sheel 2007). Incremental increases in the partial pressure of arterial carbon dioxide (PaCO_2_) have been found to contribute to increased CBF by promoting cerebral vasodilation (Ide et al. 2003). PETCO_2_ is a useful parameter that can be measured noninvasively and reflects PaCO_2_ (Brothers et al. 2009). Increased ventilation during high-intensity exercise induces a decrease in PETCO_2_, which is suggested to be a contributor to decreased CBF (Nybo et al. 2002). Oyanagi et al. (2021) used a locomotor respiratory coupling system to intentionally increase ventilation and then examined the effects of low-intensity exercise under two conditions. They found that PETCO_2_ and oxy-Hb levels were lower under the increased ventilation condition than under the normal ventilation condition, suggesting that increased ventilation during exercise may be related to the reduction in PETCO_2_ and oxy-Hb levels. Under moderate- and high-intensity water-based exercise protocols, differences in RR and PETCO_2_ may occur earlier than under land-based exercise protocols, and CBF may be lower at an earlier stage in water-based exercise protocols than in land-based exercise protocols owing to the limitation on CBF caused by vasoconstriction. However, Pugh et al. (2015) reported that CBF during low-intensity stepping exercise was higher under water conditions than land conditions. Therefore, the advantages of water-based and land-based exercise might differ depending on the intensity of exercise. In the present study, CO was higher under WC than LC at all stages, whereas PETCO_2_ and RR were higher under LC with moderate-intensity exercise. Moreover, MAP was within the autoregulation range of CBF under both WC and LC and did not differ significantly between the conditions. Importantly, oxy-Hb levels did not differ under WC and LC in most CHs in the present study. Thus, we conclude that measuring only oxy-Hb levels, which reflect neural activity, cannot explain the factors or mechanisms that produce differences in CBF under WC and LC conditions.

Although the differences in oxy-Hb levels between WC and LC were not significant in most CH at each stage, CH7 showed a significant main effect for the exercise factor, with CH7 significantly higher under WC than LC. CH7 corresponds to the left-side DLPFC; as differences were found only in this region, we speculate that functional connections exist between the DLPFC and sensory and motor cortices. Anwar et al. (2016) reported that simple tapping movements make functional connections between the PFC and the sensory and motor cortices to execute the movement. Although the actions of cycling and finger tapping differ, and other factors such as force generation capacity and rhythm formation must be considered, It is thought that the movement is formed on the basis of these. In addition to this, water immersion and water-based exercise include stimulation caused by the pressure sensation from water pressure and temperature sensation from water temperature. Sato et al. (2012) reported an increase in oxy-Hb levels in the sensory and motor cortices during water immersion up to the femur. Dalecki and Bock (2013) reported that decreased muscle tone in the arm during water immersion reduces the intrinsic sensation during isometric tasks and the passive positioning of the arm in the water. It is not clear whether the activation of the motor cortex differs during water-based and land-based exercise, but we postulate that the external information associated with immersion affects the functional connections between the sensory, motor, and prefrontal cortices, leading to differences under WC and LC conditions. Furthermore, previous studies have shown that walking in complex environments on land (e.g., obstacle walking) and posture control result in higher PFC activity than in normal conditions (Mihara et al. 2016; Maidan et al 2018). Such complex walking has also been reported to be associated with executive and attentional functions (Clark 2015). Although the exercise performed in this study was bicycle exercise rather than walking exercise, water-based exercise may activate PFC more because the movement must be performed in a complex water environment with water resistance, water currents, and buoyancy.

We acknowledge that this study has limitations. First, the sample size of this study was smaller than the number of people detected using the G*Power software. This may have affected the test results of the statistics. Future studies should consider increasing the number of participants.

Second, the study was performed using only healthy young men; therefore, we cannot apply our findings to older populations. Third, the fNIRS was attached after the participant entered the water; thus, we were not able to survey the change in oxy-Hb concentration during the environment change from land to water, which might have affected oxy-Hb levels. In studies where differences in CBF and oxy-Hb levels were found owing to differences in environmental conditions, i.e., water vs. land, the participant entered an empty tank that was later injected with water. Hence, the oxy-Hb values under the WC condition in the present study may be underestimated. Forth, previous studies have suggested that NIRS signals are affected by changes in skin blood flow and that cerebral oxygenation dynamics measured by NIRS during exercise do not accurately reflect cerebral blood flow response and cerebral metabolism (Takahashi et al. 2011; Sorensen et al. 2012). In this study, skin blood flow was not measured, the oxy-Hb values may not accurately reflect the neural activity of the PFC. Therefore, future studies should use skin blood flow measurements to investigate this issue.

In conclusion, oxy-Hb levels increased as exercise intensity was increased under WC and LC conditions. However, the advantages of increased SV and CO associated with water immersion may not affect oxy-Hb levels in the PFC. Nevertheless, oxy-Hb concentrations were higher in the WC than in the LC in some regions. Thus, it is possible that differences in motor control exist that execute movements based on information transmitted from the sensory and motor cortices.

## Data Availability

All data generated or analyzed during this study are included in this published article.
